# *Modestobacter lacusdianchii* sp. nov., a Phosphate-Solubilizing Actinobacterium with Ability to Promote *Microcystis* Growth

**DOI:** 10.1371/journal.pone.0161069

**Published:** 2016-08-18

**Authors:** Bing-Huo Zhang, Nimaichand Salam, Juan Cheng, Han-Quan Li, Jian-Yuan Yang, Dai-Ming Zha, Yu-Qin Zhang, Meng-Jie Ai, Wael N. Hozzein, Wen-Jun Li

**Affiliations:** 1 College of Life Science, Jiujiang University, Jiujiang, PR China; 2 State Key Laboratory of Biocontrol and Guangdong Provincial Key Laboratory of Plant Resources, School of Life Sciences, Sun Yat-Sen University, Guangzhou, PR China; 3 Institute of Medicinal Biotechnology, Chinese Academy of Medical Sciences and Peking Union Medical College, Beijing, PR China; 4 Bioproducts Research Chair (BRC), Zoology Department, College of Science, King Saud University, Riyadh, Kingdom of Saudi Arabia; 5 Botany and Microbiology Department, Faculty of Science, Beni-Suef University, Beni-Suef, Eqypt; Universidad de Salamanca, SPAIN

## Abstract

A novel actinobacterium, designated strain JXJ CY 19^T^, was isolated from a culture mat of *Microcystis aeruginosa* FACHB-905 collected from Dianchi Lake, South-west China. 16S rRNA gene sequences comparison of strain JXJ CY 19^T^ and the available sequences in the GenBank database showed that the strain was closely related to *Modestobacter marinus* 42H12-1^T^ (99.1% similarity) and *Modestobacter roseus* KLBMP 1279^T^ (99.0%). The isolate had *meso*-diaminopimelic in the cell wall with whole-cell sugars of mannose, rhamnose, ribose, glucose, galactose, and arabinose. The menaquinone detected was MK-9(H_4_), while the major cellular fatty acids include C_17:1_ *ω*8*c*, C_15:0_ iso, C_15:1_ iso G and C_16:0_ iso. The polar lipids were diphosphatidylglycerol, phosphatidylethanolamine, phosphatidylinositol, phosphatidylinositol mannoside and an unidentified phospholipid. The DNA-DNA hybridization values between strains JXJ CY 19^T^ and the closely related type strains *Modestobacter marinus* CGMCC 4.5581^T^ and *Modestobacter roseus* NBRC 108673^T^ were determined to be 50.8 ± 0.8% and 44.1 ± 1.7%, respectively. The DNA G+C content was 71.9 mol%. On the basis of the above taxonomic data and differences in physiological characters from the closely related type strains, strain JXJ CY 19^T^ was recognized as a novel species of the genus *Modestobacter*, for which the name *Modestobacter lacusdianchii* sp. nov. (JXJ CY 19^T^ = KCTC 39600^T^ = CPCC 204352^T^) is proposed. The type strain JXJ CY 19^T^ can solubilize calcium phosphate tribasic (Ca_3_(PO_4_)_2_), phytin and _L_-*α*-phosphatidylcholine. The phosphate-solubilizing property of the novel actinobacterium could be a possible factor for the increase in growth of *Microcystis aeruginosa* FACHB-905 in ecosystem where the amount of available soluble phosphate is limited such as Dianchi Lake.

## Introduction

Dianchi Lake, the largest freshwater lake in Yunnan Province and the sixth largest in China, has been heavily polluted owing to unchecked inflow of industrial, agricultural, and domestic wastes. The untreated disposal of waste is the leading cause for high biochemical oxygen demand, nitrate and phosphate, thereby providing a source for increase of cyanobacterial blooms [1]. The algal blooms which predominantly occur in warm season are dominated by the genus *Microcystis* [1], of which the most common one is that of *Microcystis aeruginosa* [2, 3]. The *Microcystis* mat is found to be associated with a host of other bacteria including those belonging to the phyla *Alphaproteobacteria*, *Betaproteobacteria*, *Gammaproteobacteria*, *Actinobacteria* and *Bacteroidetes* [4–7]. Co-existence between the two types of microorganisms is apparently maintained by the supply of growth factors, microelement [4], phosphate [8] and probably available carbon source (CO_2_) [9] by the bacteria to *Microcystis*, which in turn, provide organic nutrients [4, 10–12] and safer growing environment [4, 13] to the bacteria.

During analysis of culture mat of *Microcystis aeruginosa* FACHB-905 isolated from Dianchi Lake (http://algae.ihb.ac.cn/), a phosphate-solubilizing novel actinobacterium designated strain JXJ CY 19^T^ belonging to the genus *Modestobacter* was isolated. This manuscript described the polyphasic characterization of this actinobacterial strain. The manuscript also reports the effect on the growth of *M*. *aeruginosa* under *in vitro* conditions by co-culturing with the novel actinobacterial strain JXJ CY 19^T^.

## Materials and Methods

### Isolation and maintenance of strain

About 0.2 ml of *M*. *aeruginosa* FACHB-905 culture obtained from Freshwater Algae Culture Collection at the Institute of Hydrobiology (FACHB collection), Chinese Academy of Sciences (CAS), Wuhan, China was spread on International *Streptomyces* Project medium 2 (ISP 2) [14] and incubated at 28°C for 5 days. Bacterial colonies arising on the isolation media were selected and repeatedly streaked on ISP 2 agar plates to obtain pure cultures. Purified strain JXJ CY 19^T^ was maintained on ISP 2 slants at 28°C and stored as glycerol suspensions (30%, v/v) at -80°C.

### Phenotypic characteristics

Morphology was observed using light microscope (BX43; Olympus) and electron microscope (QUANTA200; FEI). Gram staining was carried out by using the standard Gram’s stain procedure. Growth at various temperatures (4–50°C), pH (4.0–11.0) and NaCl concentrations (0–10%, w/v) were examined according to method described by Xu et al. [15] using ISP 2 as the basal medium. Catalase activity was determined using H_2_O_2_ (3%). Oxidase activity was tested according to Kovacs [16]. Other phenotypic characteristics were determined according to Goodfellow [17] and Williams et al. [18]. Enzyme activities were tested by using the commercial API ZYM system (bioMérieux). Assimilation of various substrates was tested using Biolog GN III Micro Plate assays following manufacturer’s instructions.

### Chemotaxonomy

Analysis of isomer of diaminopimelic acid and whole-cell sugars were performed according to the procedures developed by Hasegawa et al. [19] and Tang et al. [20] respectively. Polar lipids were extracted according to the method described by Minnikin et al. [21] and analyzed as described by Collins & Jones [22]. Menaquinones were extracted according to the method described by Collins et al. [23] and analyzed using HPLC [24]. Analysis of fatty acids was performed by GC using the microbial identification system (Sherlock Version 6.1; MIDI database: TSBA6) [25]. Biomass for fatty acid analysis was obtained from cells grown on tryptone soy agar (TSA; Difco) at 28°C for 4 days. The G+C content of genomic DNA of strain JXJ CY 01^T^ was determined by using HPLC [26].

### Molecular analysis

16S rRNA gene sequence of strain JXJ CY 19^T^ was aligned with sequences of the most closely related taxa by using CLUSTAL_X program version 1.83 [27]. Phylogenetic trees were constructed by using the neighbour-joining [28], maximum-likelihood [29] and maximum-parsimony [30] tree-making algorithms using MEGA version 5.0 software [31]. Topologies of the phylogenetic trees were evaluated by bootstrap analysis of Felsenstein [32] with 1000 replicates. The genomic relatedness between strain JXJ CY 19^T^ and closely related strains were performed as described by Ezaki et al. [33].

### Phosphate solubilization

The ability of strain JXJ CY 19^T^ and other members of the genus *Modestobacter* to solubilize insoluble phosphate were determined on plates using phosphate-solubilizing media [glucose, 10 g; (NH_4_)_2_SO_4_, 0.5 g; MgSO_4_·7H_2_O, 0.3 g; NaCl, 0.3 g; KCl, 0.3 g; FeSO_4_·4H_2_O, 0.036 g; MnSO_4_·4H_2_O, 0.03 g; Ca_3_(PO_4_)_2_, 10 g or _L_-*α*-phosphatidylcholine, 2.0 g or phytin, 2.0 g; distilled water, 1000 ml; pH 7.0] [34]. Since some microorganisms can solubilize insoluble phosphates in liquid medium despite showing no clear phosphate-solubilizing zone on agar plates [35], the phosphate-solubilizing ability is further confirmed using liquid cultures. Cultures of the tested strains grown in ISP 2 broth (28°C, 2–5 days) were centrifuged (4,860×*g*, 20 min, 4°C), and the biomass resuspended in small aliquots of sterilized distilled water. Cell suspension was inoculated into the phosphate-solubilizing media with a final cell density of 1×10^6^ CFU/ml. For the control, the bacterial cell inoculum was replaced with sterile water. Culture broth was centrifuged (4,860×*g*, 20 min) on the 7^th^ day of incubation, and the amount of available phosphorus in the supernatant (measured as phosphate equivalent) determined colorimetrically using standard protocol as described below [34].

Reaction mixtures containing 5 ml supernatant, 0.1 ml 2,4-dinitrophenol solution (0.011 M) and 5 ml Mo-Sb reagent solution were adjusted to a final volume of 50 ml with distilled water, briefly mixed and kept incubated at 20°C for 30 min. Absorbance of the reaction mixture was monitored at 700 nm, and the available phosphorus in each reaction mixture determined against a standard curve of potassium phosphate. The Mo-Sb reagent solution contained (per liter) sulfuric acid, 2.87 mol; ammonium molybdate, 8.1 mmol; antimonyl potassium tartrate, 1.5 mmol; ascorbic acid, 85.2 mmol (added into the solution just before use).

Concentration of the available phosphorus in the culture media was calculated based on the following equation:

$$X=\frac{\left(P\times V1\times K\right)}{V2}$$

        where X represent available phosphorus in the culture media,

        P, available phosphorus in the reaction mixture,

        V1, total volume of reaction mixture,

        V2, volume of culture supernatant added in the reaction mixture, and

        K, the dilution ratio used for measuring the absorbance.

### Effect on the growth of *M*. *aeruginosa* by co-culturing with strain JXJ CY 19^T^ under *in vitro* condition

As *M*. *aeruginosa* FACHB-905 culture mat has many associated bacteria, the culture FACHB-905 is purified prior to co-culture for understanding the effects of strain JXJ CY 19^T^ on its growth. 0.1 ml *M*. *aeruginosa* FACHB-905 culture mat was spread on HGZ agar medium [36] and kept incubated under illumination of 30–50 μmol photon/m^2^/s on a 12-h light/dark cycle at 25°C. The cultures were incubated until green colonies were observed. These colonies were checked for bacterial contamination by spreading on ISP 2 agar, with parallel observation under light microscope. Absence of bacterial growth on ISP 2 indicated a pure *M*. *aeruginosa* culture preparation. Pure colonies were further inoculated into fresh HGZ media, and incubated for another 30 days.

Biomass of purified *M*. *aeruginosa* FACHB-905 were collected by centrifugation (4,860×*g*, 20 min, 4°C) and inoculated into HGZ and modified HGZ media (KH_2_PO_4_ replaced with Ca_3_(PO_4_)_2_ or _L_-*α*-phosphatidylcholine) with an initial density of approximately 2×10^6^ CFU/ml. The media were then co-inoculated with strain JXJ CY 19^T^ corresponding to inoculum densities of 0.2×10^7^ CFU/ml, 1×10^7^ CFU/ml and 5×10^7^ CFU/ml. For the control, bacterial cell suspension was replaced by sterilized water. The co-cultures were kept incubated under illumination of 30–50 μmol photon/m^2^/s in a 12-h light/dark cycle. Cells of *M*. *aeruginosa* were counted under light microscope (Olympus BX43, Japan) on the 7^th^ and 60^th^ days of incubation and while for strain JXJ CY 19^T^, plate colony counting method was adopted to determine the CFU. The available phosphorus in the modified HGZ media was also concurrently measured.

### Statistical analysis

All the experiments for phosphate solubilization and determination of CFU were done in triplicates, and the values were expressed as their mean. These data were subjected to one-way ANOVA at *P* < 0.05 and *P* < 0.01 using SPSS 17 software (SPSS Inc).

## Results

### Phenotypic characteristics

Strain JXJ CY 19^T^ was Gram-stain positive and non-endospore-forming. Cells of strain JXJ CY 19^T^ were short rods (straight or lightly curved) with size of 0.5–1 × 1.0–2.5 μm when cultivated in ISP 2 broth for less than 24 hours. The cells gradually turned coccoid in the later stages. Strain JXJ CY 19^T^ could grow at 4–40°C, pH 6.0–9.0 and 0–6% (w/v) NaCl, with optimal growth at 25–28°C, pH 7.0–8.0 and 0–3% (w/v) NaCl. The isolate was found positive for catalase and oxidase tests.

Detailed phenotypic characteristics of the strain are given in Table 1 and species description.

**Table 1:** Comparative characteristics between strain JXJ CY 19^T^ and other members of the genus *Modestobacter*.

	1	2	3	4^[^^37^^]^*	5^[^^38^^]^*	6^[^^38^^]^*	7^[^^39^^]^*
Colony colour on ISP 2 medium	Pink	Pink-deep orange to dark	Pink	Skin-colour to pale pink	Light-dark orange	Light orange-pink	Pink-deep orange to dark
**G+C content (mol %)**	71.9	72.3*	71.7*	69.9	72	71.5	73
**Major fatty acids (>10%)**	C_15:0_ iso, C_16:0_ iso, C_15:1_ iso G, C_17:1_ *ω*9*c*	C_16:0_ iso, C_16:0_*, C_17:1_ *ω*9c*, C_17:0_*	C_16:0_ iso, C_17:1_ *ω*9*c*, C_15:0_ iso*	C_15:0_ iso, C_16:0_ iso	C_16:0_ iso, C_15:0_ iso, C_16:1_ *ω*9*c*	C_17:1_ *ω*9*c*, C_17:0_, C_16:0_ iso	C_16:0_ iso, C_15:0_ iso
**Polar lipids**	DPG, PE, PI, PIM, PL	DPG, PE, PG, PI, APL*	DPG, PE, PI, PIM, APL, PL*	DPG, PE, PI	DPG, PE, PG, PI, PIM	DPG, PE, PG, PI, PIM	DPG, PG, PE, PI
**Whole-cell sugars**	Ara, Gal, Glu, Man, Rha, Rib	ND	Gal, Glu, Rib*	Gal, Glu, Rib	Ara, Gal, Glu, Rib	Gal, Glu, Rib	Gal, Glu, Rib
**H**_**2**_**S**	-	+	-	-	ND	ND	-
**NaCl range (w/v, %)**	0–6	0–6	0–9	0–8	0–8	0–8	0–3
**Growth temperature range (°C)**	4–40	4–40	10–40	0–28	20–37	20–37	4–30
**pH range for growth**	6–9	7–9	6–9	3–12	6–9	5–9	5–9
**Nitrate reduction**	-	+	-	+	+	+	+
**Urease activity**	-	-	-	ND	+	+	+
**Utilization of**							
_**D**_**-Arabinose**	-	+	+	ND	ND	ND	-
_**D**_**-(+)-Cellobiose**	+	-	+	-	+	-	+
_**D**_**-Glycerol**	+	-	w	+	-	-	+
**Maltose**	+	+	+	w	+	-	+
**Lactose**	-	-	+	+	-	-	+
***Myo*-inositol**	-	-	-	-	-	-	+
_**D**_**-Galactose**	+	+	+	+	-	-	-
**Mannitol**	+	+	+	+	ND	ND	+
**Salicin**	-	-	+	+	-	-	+
_**D**_**-Mannose**	+	+	-	w	-	-	+
_**L**_**-Rhamnose**	+	+	w	w	-	-	+
_**D**_**-Sorbitol**	+	+	w	-	+	-	-
_**D**_**-Melibiose**	+	-	+	-	+	-	+
**Sodium acetate**	+	-	-	+	ND	ND	-
**Citric acid**	+	-	-	-	+	-	-
**Malate**	-	+	+	+	+	+	+
_**L**_**-Alanine**	+	+	-	-	-	-	+
_**L**_**-Glycine**	+	-	-	ND	ND	ND	-
_**L**_**-Phenylalanine**	+	+	-	ND	ND	ND	-
**Dextrin**	+	-	w	-	+	+	-
_**D**_**-Fucose**	-	+	w	+	-	-	+
**Inosine**	+	-	w	+	+	-	+

1, JXJ CY 19; 2, *M*. *marinus* CGMCC 4.5581^T^; 3, *M*. *roseus* NBRC 108673^T^; 4, *M*. *multiseptatus* AA-826^T^; 5, *M*. *lapidis* MON 3.1^T^; 6, *M*. *muralis* MDVD1^T^; 7, *M*. *versicolor* DSM 16678^T^.

+: positive; -: negative; w: weakly; ND: no determined.

Data for strains JXJ CY 19, *M*. *marinus* CGMCC 4.5581^T^, *M*. *roseus* NBRC 108673^T^, except those marked by *asterisks*, are from this study.

DPG: diphosphatidylglycerol; PG: phosphatidylglycerol; PE: phosphatidylethanolamine; PI: phosphatidylinositol; PIM: phosphatidylinositol mannosides; APL, unidentified aminophospholipid(s); PL, unidentified phospholipid(s); L, unidentified lipid(s).

Ara: arabinose; Gal: galactose; Glu: glucose; Man: mannose; Rha: rhamnose; Rib: ribose.

### Chemotaxonomy

Strain JXJ CY 19^T^ contained *meso*-DAP, along with mannose, rhamnose, ribose, glucose, galactose, and arabinose in the whole-cell hydrolysates. Polar lipids consisted of diphosphatidylglycerol, phosphatidylethanolamine, phosphatidylinositol, phosphatidylinositol mannosides and an unidentified phospholipid (S1 Fig). The menaquinone detected was MK-9 (H_4_). The fatty acids profile was C_15:0_ iso (25.4%), C_16:0_ iso (25.3%), C_15:1_ iso G (10.2%), C_17:1_ *ω*9*c* (9.9%), summed feature 3 comprising C_16:1_ *ω*6*c* and/or C_16:1_ *ω*7*c* (4.4%), C_17:0_ iso (3.7%), summed feature 9 comprising C_16:0_ 10-methyl (3.1%), C_16:0_ (3.0%), C_16:1_ iso H (2.6%), C_17:0_ (1.4%) and C_15:1_ *ω*6*c* (1.2%). The G+C content of the genomic DNA was determined to be 71.9 mol%.

### Molecular analysis

**Fig 1:**
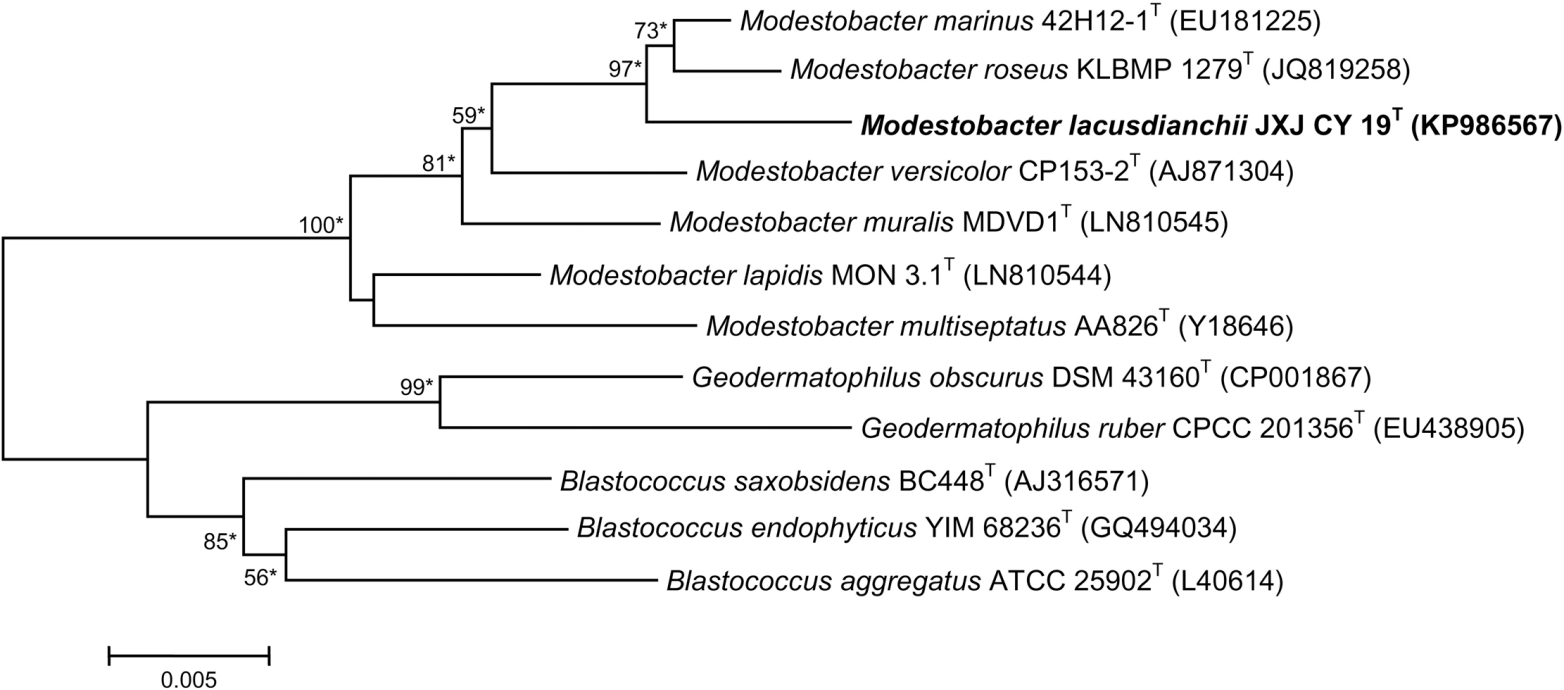
Neighbour-joining phylogenetic tree based on 16S rRNA gene sequences of strain JXJ CY 19^T^ and representative type strains of the family *Geodermatophilaceae*. Bootstrap values (expressed as percentages of 1,000 replications) of above 50% are shown at the nodes. *Asterisks* indicate clades that were conserved using the maximum-parsimony and maximum-likelihood methods. Bar, 0.005 sequence divergence.

Strain JXJ CY 19^T^ showed highest 16S rRNA gene sequence similarities with members of the genus *Modestobacter*: *Modestobacter marinus* 42H12-1^T^, 99.12%; *M*. *roseus* KLBMP 1279^T^, 99.03%; *M*. *versicolor* CP 153-2^T^, 98.41%; *M*. *muralis* MDVD1^T^, 98.21%; *M*. *lapidis* MON 3.1^T^, 97.99% and *M*. *multiseptatus* AA-826^T^, 97.64%. The strain formed a stable clade with strains *M*. *marinus* 42H12-1^T^ and *M*. *roseus* KLBMP 1279^T^ in the phylogenetic dendrograms based on 16S rRNA gene sequences (Fig 1; S2 and S3 Figs), indicating that the strain belongs to the genus *Modestobacter*. Based on the analysis of the 16S rRNA gene sequences, the phylogenetic trees and the recommendation of Stackebrandt and Ebers [40], the two strains *M*. *marinus* CGMCC 4.5581^T^ and *M*. *roseus* NBRC 108673^T^ were considered for DNA-DNA relatedness study. The DNA-DNA hybridization values between strain JXJ CY 19^T^ and type strains *M*. *marinus* CGMCC 4.5581^T^ and *M*. *roseus* NBRC 108673^T^ were determined to be 50.81 ± 0.84% and 44.07 ± 1.66% respectively (S1 Table).

In addition to the differences in the genomic DNA relatedness, strain JXJ CY 19^T^ could be differentiated from the type strains of the genus *Modestobacter* by the characteristics listed in Table 1. Based on the data in this study, we propose that strain JXJ CY 19^T^ represents a novel species of the genus *Modestobacter*, for which the name *Modestobacter lacusdianchii* sp. nov. is proposed.

### Description of *Modestobacter lacusdianchii* sp. nov.

*Modestobacter lacusdianchii* sp. nov. (la.cus.di.a'n.chii L. gen. n. lacus, of a lake; N.L. gen. n. dianchii, of Dianchi; N.L. gen. n. *lacusdianchii*, of Dianchi lake).

Cells are aerobic, Gram-staining positive, non-spore-forming, short rods (0.5–1.0 × 1.0–2.5 μm, straight or lightly curved), or cocci with a tendency to aggregate. Colonies are pink throughout growth. Growth is observed at 4–40°C, pH 6.0–9.0 and 0–6% (w/v) NaCl, with optimal growth at 25–28°C, pH 7.0–8.0 and 0–3% (w/v) NaCl. Utilizes _D_-(+)-cellobiose, _D_-fructose, _D_-galactose, _D_-glucose, _D_-glycerol, myo-inositol, _D_-mannitose, _D_-mannose, _D_-raffinose, _D_-sorbitol, _L_-rhamnose, sucrose, _D_-trehalose, _D_-xylose, _D_-melibiose, sodium acetate, _L_-lactose and dulcitol as sole carbon sources, but not _D_-arabinose, _D_-ribose, _D_-xylitol or sodium propionate. Utilizes _L_-alanine, _L_-arginine, _L_-aspartic acid, _L_-cysteine, _L_-glutamic acid, _L_-glutamine, glycine, _L_-leucine, _L_-isoleucine, _L_-methionine, _L_-lysine, _L_-phenylalanine, _L_-valine, _L_-threonine, _L_-tyrosine, _L_-proline, _L_-tryptophan, _L_-serine and hypoxanthine as sole nitrogen sources, but not _L_-histidine. Positive for catalase, oxidase and phosphatase assays, but negative for milk coagulation, milk peptonization, nitrate reduction, methyl red test, Voges-Prokauer test, H_2_S production, and hydrolysis of casein, gelatin, cellulose and Tweens 20, 40, 60 and 80. Acid is produced from starch, aesculin and _D_-tagatose (API 50 CH). Cell-wall peptidoglycan contains *meso*-DAP, with mannose, rhamnose, ribose, glucose, galactose, and arabinose as whole-cell sugars. Polar lipids consist of phosphatidylglycerol, phosphatidylethanolamine, phosphatidylinositol and phosphatidylinositol mannosides. The menaquinone is MK-9(H_4_). Major fatty acids are C_17:1_ *ω*8*c*, C_15:0_ iso, C_15:1_ iso G and C_16:0_ iso. The G+C content of the genomic DNA of the type strain is determined to be 71.9 mol%.

The type strain, JXJ CY 19^T^ (= KCTC 39600^T^ = CPCC 204352^T^), was isolated from the culture mat of *Microcystis aeruginosa* FACHB-905 collected from Dianchi Lake, China. The 16S rRNA gene sequence of strain JXJ CY 19T has been deposited in GenBank under the accession number KP986567.

### Phosphate solubilization

All the seven tested strains formed no visible halo zones for solubilization of either Ca_3_(PO_4_)_2_ or _L_-*α*-phosphatidylcholine in plates, while strains *M*. *marinus* CGMCC 4.5581^T^, *M*. *roseus* NBRC 108673^T^, *M*. *versicolor* CP 153-2^T^ and *M*. *muralis* MDVD1^T^ formed weak zone for solubilization of phytin. Under liquid culture assay, all the strains could solubilize _L_-*α*-phosphatidylcholine and phytin with a detection of additional available phosphorus of 0.5–0.9 mg/l and 0.5–4.5 mg/l respectively than the control. Only strain JXJ CY 19^T^ and *M*. *muralis* MDVD1^T^ were able to solubilize Ca_3_(PO_4_)_2_ with an available phosphorus content of 0.2–0.3 mg/l higher than that of the control

### Effect on the growth of *M*. *aeruginosa* FACHB-905 by co-culturing with strain JXJ CY 19^T^ under *in vitro* condition

Cell density of *M*. *aeruginosa* FACHB-905 in the control media increased from initial 2×10^6^ CFU/ml at day 0 to 9.03×10^6^ CFU/ml and 9.87×10^7^ CFU/ml on day 7 and 60 respectively in the absence of bacterial co-inoculant. Co-culturing with 0.2×10^7^ CFU/ml of JXJ CY 19^T^ enhanced the cell density of *M*. *aeruginosa* from the control by 10.29% and 12.06% (*P* < 0.05) on day 7 and day 60 respectively (Tables 2 and 3). However, with increased bacterial inoculum density (5×10^7^ CFU/ml), cyanobacterial cell growth were initially observed to decrease on day 7 but recovered significantly on day 60, as compared to control at *P* < 0.01 (Tables 2 and 3).

**Table 2:** Cell numbers (mean±standard deviation; n = 3) of *Microcystis aeruginosa* FACHB-905 after co-culturing with strain JXJ CY 19^T^ for 7 days.

Cell density of strain JXJ CY 19^T^ (×10^7^ CFU/ml)		Cell density of FACHB-905 (×10^6^ CFU/ml)		
Initial	Final	HGZ media	a	b
0	0	9.03±0.33	6.85±0.31	6.72±0.42
0.2	~0.1	9.96±0.45*	7.45±0.36	8.90±0.38**
1.0	~0.3	8.70±0.39	8.13±0.51*	9.15±0.49**
5.0	~1.0	7.75±0.24**	9.15±0.41**	9.60±0.44**

Statistical comparisons with the controls were made using ANOVA (* *P* < 0.05, ** *P* < 0.01).

a and b represented KH_2_PO_4_ in HGZ media was replaced with Ca_3_(PO_4_)_2_ and _L_-*α*-phosphatidylcholine, respectively.

**Table 3:** Cell numbers (mean±standard deviation; n = 3) of *Microcystis aeruginosa* FACHB-905 after co-culturing with strain JXJ CY 19^T^ for 60 days.

Cell density of strain JXJ CY 19^T^ (×10^7^ CFU/ml)		Cell density of FACHB-905 (×10^6^ CFU/ml)		
Initial	Final	HGZ media	a	b
0	0	9.87±0.32	5.58±0.22	6.38±0.37**
0.2	~0.03	11.06±0.44*	11.36±0.58**	10.21±0.46**
1.0	~0.06	12.26±0.62**	17.56±0.90**	11.95±0.54**
5.0	~0.30	18.53±0.88**	18.03±0.92**	12.41±0.40**

Statistical comparisons with the controls were made using ANOVA (* *P* < 0.05, ** *P* < 0.01).

a and b represented KH_2_PO_4_ in HGZ media was replaced with Ca_3_(PO_4_)_2_ and _L_-*α*-phosphatidylcholine, respectively.

**Fig 2:**
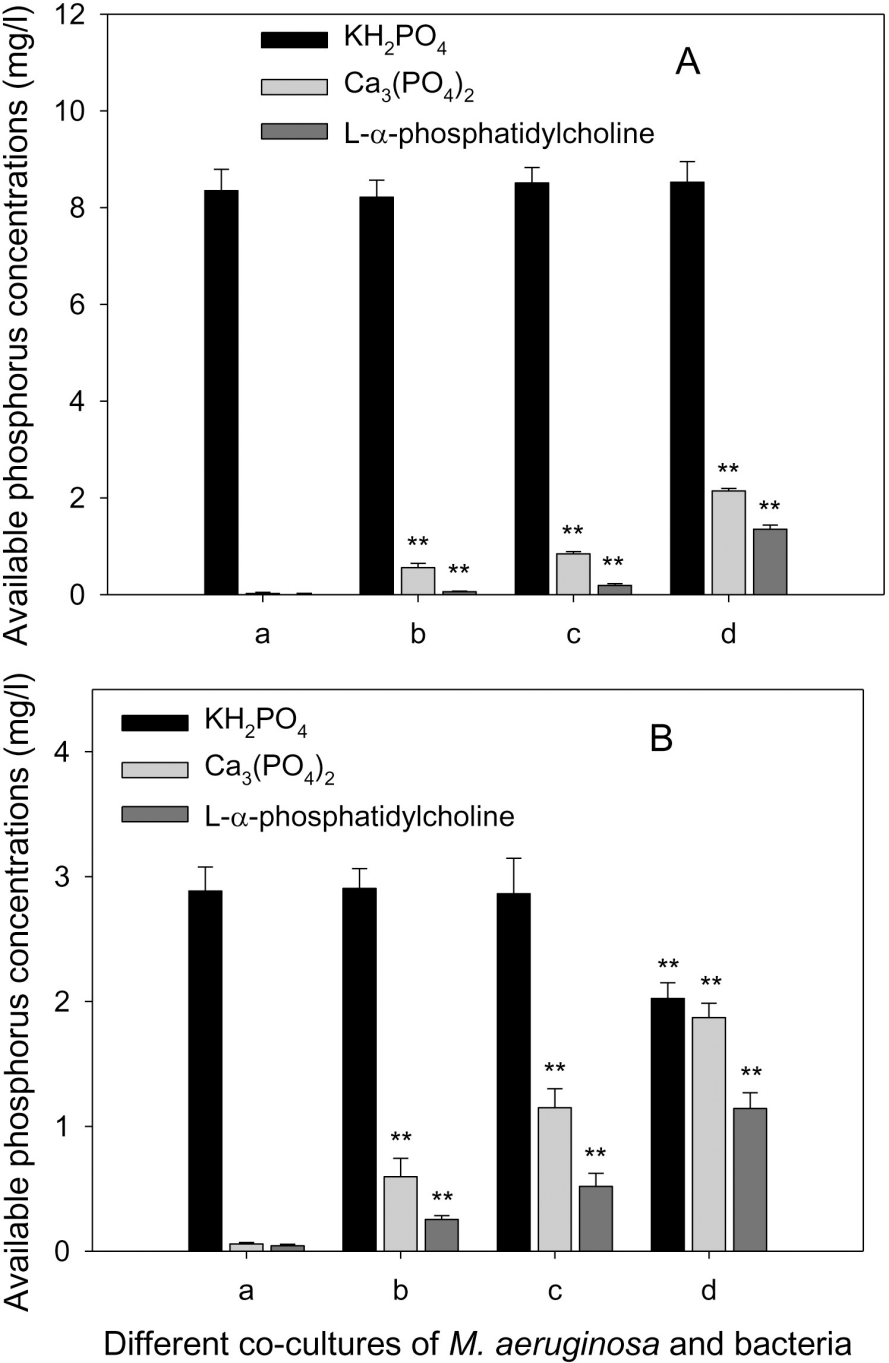
**Available phosphorus concentrations of different co-cultures of *M*. *aeruginosa* with strain JXJ CY 19**^**T**^ **in modified HGZ media on day 7 (A) and 60 (B).** a, b, c, and d represented the treatments of initial bacterial cell densities of 0×10^7^ CFU/ml, 0.2×10^7^ CFU/ml, 1×10^7^ CFU/ml and 5×10^7^ CFU/ml respectively. Statistical comparisons with the control were made using ANOVA (* *P* < 0.05, ** *P* < 0.01).

When KH_2_PO_4_ in the medium is replaced either by Ca_3_(PO_4_)_2_ or _L_-*α*-phosphatidylcholine, the increase in cyanobacterial cell density is quite significant (*P* < 0.05, *P* < 0.01), and become more prominent with increase in inoculum density of strain JXJ CY 19^T^ (Tables 2 and 3). This increase may be accounted for by the solubilization of the insoluble phosphate (Fig 2; *P* < 0.01).

## Discussion

Many studies have been conducted to understand the phytoplankton communities of eutrophic lakes [1, 4]. It was usually reported that algae especially *Microcystis* dominate this communities [1–4]. In a similar study by Parveen et al. [7], *Microcystis* colonies appeared to be depleted of *Actinobacteria*, while enriched in *Gammaproteobacteria*. In our earlier studies [36, 41], we have found the presence of antialgal compounds from a novel *Streptomyces jiujiangenesis* strain JXJ 0074^T^ [42]. This might be another reason for a non-cohesive existence of free living *Actinobacteria* in the phytoplankton communities of eutrophic lakes as reported by Parveen et al. [7]. In contrast to the above studies, the present study indicates the presence of a growth-promoting actinobacteria of the genus *Modestobacer* within these lake bacterial communities.

The genus *Modestobacter* belongs to the family *Geodermatophilaceae* [43, 44] of the order *Geodermatophilales* [45]. Like other strains of the order *Geodermatophiles*, the genus *Modestobacter* tend to be associated with extreme biomes, including deteriorated sandstone [38], desert plateau [39], deep-sea sediment [46] and coastal halophytes [47]. The present study reports the isolation of a *Modestobacter* strain from a eutrophic lake located in Yunnan, China. Interestingly all the *Modestobacter* strains were found to solubilize insoluble phosphorus despite differences in their origins.

Phosphorus is a key chemical element essential for biological activities. Only 5–8% of the total phosphorus in the water was available to biology [48] and is, therefore, considered as the principal limiting nutrient for algal growth in most freshwater habitats [49, 50]. *Microcystis* mat are often found associated with many bacteria in a complex relationship. Among these bacteria isolated from different freshwater cyanobacterial mat, the strains *Pseudomonas sp*. X, *Erythrobacter* sp. Y6, *Gordonia* sp. txj1302RI and *Burkholderia* sp. txj1302Y4 have been found to decompose the insoluble phosphate and thereby making it available for the growth of *Microcystis* [8, 51–53]. Similar result is found during the present study The novel strain JXJ CY 19^T^ was found to solubilize inorganic and organic phosphate from insoluble source (Fig 2) under *in vitro* condition, and this soluble phosphorus are made available for growth of *M*. *aeruginosa* FACHB-905 (Tables 2 and 3).

In addition to the availability of phosphorus source, additional factors might also be responsible for the increase in growth of cyanobacteria. This is indicated by the fact that despite adequate phosphorus in the normal HGZ media (Fig 2), co-culturing with lower cell density of strain JXJ CY 19^T^ result in significant increase in growth of *M*. *aeruginosa* (Tables 2 and 3). Similar findings have been reported by several studies of cyanobacterial mat-associated bacteria. Zhao et al. [8] found that *Actinobacteria* strain *Gordonia* sp. txj1302RI produce unknown substances that promote the growth of *Microcystis*. de-Bashan et al. [54] reported that *Azospirillum* spp. produce indole-3-acetic acid that helps in promoting the growth of *Chlorella vulgaris*. The symbiotic relationship of *M*. *aeruginosa* is also influenced by the cellular density of the associated bacteria. Under condition of abundant phosphorus, lower cell density (< 0.2×10^7^ CFU/ml) of strain JXJ CY 19^T^ stimulate the growth of *M*. *aeruginosa* FACHB-905 while and higher cell density (~1–5×10^7^ CFU/ml) inhibit its growth (Table 2). With time, the cell densities of *M*. *aeruginosa* recuperate, but with a concomitant decrease in the cell density of strain JXJ CY 19^T^.

## Supporting Information

S1 Fig Two-dimensional thin-layer chromatogram of polar lipids of strain JXJ CY 19^T^ stained with 5% ethanolic molybdophosphoric acid. The chromatographic conditions were as follows: Silica Gel 60 thin-layer plates (10 by 10 cm) were spotted with 10 μl of a whole-cell lipid extract. Chloroform-methanol-water (65:25:4, v/v/v) was used to develop the chromatogram in the first direction, and chloroform-acetic acid-methanol-water (80:18:12:5, v/v/v/v) was used in the second direction. DPG, diphosphatidylglycerol; PE, phosphatidylethanolamine; PI, phosphatidylinositol; PIM, phosphatidylinositol mannosides; PL, unidentified phospholipid.
(PDF)

S2 Fig Maximum-Parsimony phylogenetic tree based on 16S rRNA gene sequences of strain JXJ CY 19^T^ and representative type strains of the family *Geodermatophilaceae*. Bootstrap values (expressed as percentages of 1000 replications) of above 50% are shown at the branch points.
(PDF)

S3 Fig Maximum-Likelihood phylogenetic tree based on 16S rRNA gene sequences of strain XJ CY 19^T^ and representative type strains of the family *Geodermatophilaceae*. Bootstrap values (expressed as percentages of 1000 replications) of above 50% are shown at the branch points. Bar, 0.005 sequence divergence.
(PDF)

S1 Table DNA–DNA relatedness between strain JXJ CY 19^T^ and closely related members of the genus *Modestobacter*. A, JXJ CY 19^T^; B, *M*. *marinus* CGMCC 4.5581^T^; C, *M*. *roseus* NBRC 108673^T^.
(PDF)
