# Supplementary material for: Modestobacter lacusdianchii sp. nov., a Phosphate-Solubilizing Actinobacterium with Ability to Promote Microcystis Growth
Source: PLoS One. 2016 Aug 18;11(8):e0161069. doi: 10.1371/journal.pone.0161069 (PMC4990248; doi:10.1371/journal.pone.0161069)
Supplement: S1 Fig — The chromatographic conditions were as follows: Silica Gel 60 thin-layer plates (10 by 10 cm) were spotted with 10 μl of a whole-cell lipid extract. Chloroform-methanol-water (65:25:4, v/v/v) was used to develop the chromatogram in the first direction, and chloroform-acetic acid-methanol-water (80:18:12:5, v/v/v/v) was used in the second direction. DPG, diphosphatidylglycerol; PE, phosphatidylethanolamine; PI, phosphatidylinositol; PIM, phosphatidylinositol mannosides; PL, unidentified phospholipid. (PDF) [file pone.0161069.s001.pdf]

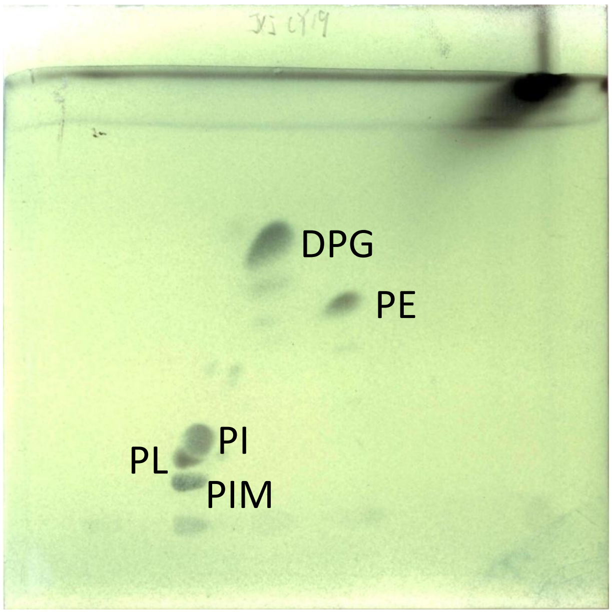

S1 Fig. Two-dimensional thin-layer chromatogram of polar lipids of strain JXJ CY 19^{T} stained with 5 % ethanolic molybdophosphoric acid. The chromatographic conditions were as follows: Silica Gel 60 thin-layer plates (10 by 10 cm) were spotted with 10 µl of a whole-cell lipid extract. Chloroform-methanol-water (65:25:4, vol/vol/vol) was used to develop the chromatogram in the first direction, and chloroform-acetic acid-methanol-water (80:18:12:5, vol/vol/vol/vol) was used in the second direction. DPG, diphosphatidylglycerol; PE, phosphatidylethanolamine; PI, phosphatidylinositol; PIM, phosphatidylinositol mannosides; PL, unidentified phospholipid.
