# Supplementary material for: Modestobacter lacusdianchii sp. nov., a Phosphate-Solubilizing Actinobacterium with Ability to Promote Microcystis Growth
Source: PLoS One. 2016 Aug 18;11(8):e0161069. doi: 10.1371/journal.pone.0161069 (PMC4990248; doi:10.1371/journal.pone.0161069)
Supplement: S3 Fig — Bootstrap values (expressed as percentages of 1000 replications) of above 50% are shown at the branch points. Bar, 0.005 sequence divergence. (PDF) [file pone.0161069.s003.pdf]

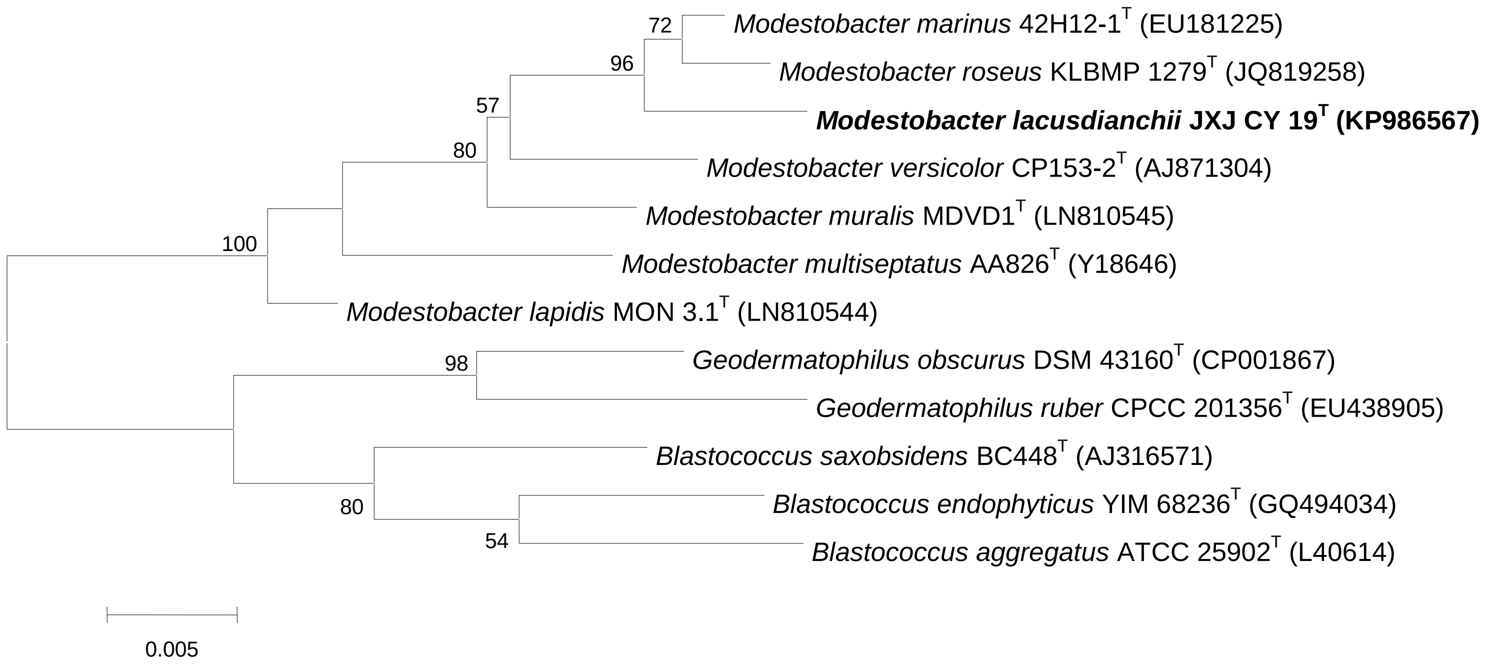

S3 Fig. Maximum-Likelihood phylogenetic tree based on 16S rRNA gene sequences of strain JXJ CY 19T and representative type strains of the family *Geodermatophilaceae*. Bootstrap values (expressed as percentages of 1000 replications) of above 50 % are shown at the branch points. Bar, 0.005 sequence divergence.
