# Supplementary material for: Modestobacter lacusdianchii sp. nov., a Phosphate-Solubilizing Actinobacterium with Ability to Promote Microcystis Growth
Source: PLoS One. 2016 Aug 18;11(8):e0161069. doi: 10.1371/journal.pone.0161069 (PMC4990248; doi:10.1371/journal.pone.0161069)
Supplement: S1 Table — A, JXJ CY 19T; B, M. marinus CGMCC 4.5581T; C, M. roseus NBRC 108673T. (PDF) [file pone.0161069.s004.pdf]

S1 Table. DNA–DNA relatedness between strain JXJ CY 19^T and closely related members of the genus *Modestobacter*. A, JXJ CY 19^T; B, *M. marinus* CGMCC 4.5581^T; C, *M. roseus* KLBMP 1279^T.

The two DNAs for DNA-DNA hybridization	DNA as probe	DNA immobilized	Values of four replicates (%)				Average (%)	The result of DNA-DNA hybridization (%)
			1	2	3	4		
A and B	A	B	55.36	53.51	51.12	54.19	53.54 ±1.79	50.81 ±0.84
	B	A	45.71	47.31	49.33	49.92	48.07 ±1.93	
A and C	A	C	40.22	46.63	43.74	48.57	44.79 ±3.64	44.07 ±1.66
	C	A	44.87	43.92	41.73	42.83	43.34 ±1.36	
